# Treatment of Dysentery

**Published:** 1852-12

**Authors:** 


*Treatment of Dysentery.*— Prof. Yandell, editor of the Western Journal of Med. and Surgery, in an editorial article contained in the Oct. No. of the Journal just named, says: "In conversing with our medical friends, we find that the opiate practice is that which has given most satisfaction in this complaint (Dysentery) during the past season. The opiate has been variously combined, as with sulphate of magnesia, turpentine, calomel, and astringents; but in the practice of all, an opiate has entered into the treatment. Opium, in some of its forms, has unquestionably been the efficient agent. In our judgment, next to opium, the saline purgatives have proved most beneficial. We have not seen calomel used with advantage in any case."

